# Circulating Extracellular Vesicles Are Strongly Associated With Cardiovascular Risk Markers

**DOI:** 10.3389/fcvm.2022.907457

**Published:** 2022-05-26

**Authors:** Ruihan Zhou, Esra Bozbas, Keith Allen-Redpath, Parveen Yaqoob

**Affiliations:** Hugh Sinclair Unit of Human Nutrition, Department of Food and Nutritional Sciences, University of Reading, Reading, United Kingdom

**Keywords:** blood lipids, blood pressure, cardiovascular risk markers, extracellular vesicles, thrombin

## Abstract

**Background:**

Extracellular vesicles (EVs) are submicron membrane-bound vesicles released from various cells, which are emerging as a potential novel biomarker in cardiovascular diseases (CVDs) due to their procoagulatory and prothrombotic properties. However, there is little information about the relationships between circulating EVs and conventional and thrombogenic risk markers of CVDs.

**Objective:**

To investigate the relationships between circulating EVs, conventional cardiovascular risk markers and thrombogenic markers in subjects with moderate risk of CVDs.

**Design:**

Subjects (*n* = 40) aged 40-70 years with moderate risk of CVDs were recruited and assessed for body mass index, blood pressure and plasma lipid profile, as well as platelet aggregation, clot formation, thrombin generation and fibrinolysis. Numbers of circulating EVs were assessed by Nanoparticle Tracking Analysis and flow cytometry. A range of assays were used to assess the procoagulatory activity of plasma and circulating EVs.

**Results:**

Circulating EV numbers were positively associated with body mass index, blood pressure, plasma triacylglycerol concentration and overall CVD risk. Higher circulating EV numbers were also associated with increased thrombin generation and enhanced clot formation, and EVs isolated from subjects with moderate CVD risk promoted thrombin generation *ex vivo*. Higher numbers of endothelial-derived EVs were associated with a greater tendency for clot lysis. Plasma triacylglycerol concentration and diastolic blood pressure independently predicted circulating EV numbers, and EV numbers independently predicted aspects of thrombin generation and clot formation and 10-year CVD risk.

**Conclusion:**

Circulating EVs were strongly associated with both conventional and thrombogenic risk markers of CVDs, and also with overall CVD risk, highlighting a potentially important role for EVs in CVDs.

## Introduction

Cardiovascular diseases (CVDs) are the leading cause of mortality worldwide (1). Smoking, hypertension, hyperlipidemia, obesity and diabetes are well established “conventional” risk factors for CVDs (2–4). However, these factors do not fully predict the risk of cardiovascular events and treatments that are highly effective in altering a single risk factor reduce, but do not eliminate, CVD risk (5–7). In this regard, there is an interest in the discovery of new risk markers to improve the assessment of CVD risk beyond the current risk stratification (8).

Increased thrombogenic activity, including greater platelet reactivity (9), increased thrombin generation (10), enhanced clot formation (11), and altered clot fibrinolysis (12), is suggested to contribute to the development of atherosclerosis and increased risk of CVDs. Extracellular vesicles (EVs) are a heterogeneous group of submicron membrane-bound particles released into extracellular spaces by almost all cells under both physiological and pathological conditions, and are therefore present in all biological fluids (13, 14). EVs carry bioactive cargo reflective of their cell of origin, which includes proteins, lipids and nucleic acids, and are mediators of cell-to-cell communication in many biological processes (13–15). They have procoagulatory and prothrombotic properties by virtue of the phospholipids and bioactive molecules on their surface and also in their internal cargo and as such, are increasingly being investigated as novel biomarkers of CVDs (16–18). There is some evidence that elevated numbers of EVs are associated with cardiovascular events (16, 19, 20), and with conventional cardiovascular risk factors, such as obesity (21, 22), hypertension (23, 24), and hyperlipidemia (20, 25). However, the nature of the relationship between circulating EVs and cardiovascular risk markers, particularly thrombogenic risk markers, is not clear. The aim of this study was to explore the hypothesis that elevated numbers of procoagulatory circulating EVs are related to increased thrombogenic activity and CVD risk.

## Materials and Methods

The analysis described in this paper is part of a larger study (clinicaltrials.gov: NCT03203512) conducted at the Hugh Sinclair Unit of Human Nutrition, School of Chemistry, Food and Pharmacy, University of Reading, in accordance with guidelines detailed in the Declaration of Helsinki, and approved by the University of Reading Research Ethics Committee (UREC 17/18). Written informed consent was obtained from each participant after explaining the study to them.

### Subjects

A total of 40 subjects (aged 40–70 years), who were non-smokers, not underweight, anemic, or hyperlipidemic and were free of kidney and liver dysfunction, CVDs and other diseases were recruited by using the Hugh Sinclair Unit of Human Nutrition volunteer database, emailing staff and students of the University of Reading and also members of local community groups and large local organizations and companies, advertising in local newspapers and websites, and distributing posters and leaflets in public places. Further exclusion criteria included prescribed medication or supplementation, consumption of alcohol above a threshold of 21 units/week (male) or 15 units/week (female), or intense regular aerobic exercise, defined as more than 20 min more than three times per week. Subjects were screened for moderate risk of CVDs (defined as 10–20% risk of a heart attack or stroke in the next 10 years) using the QRISK2 scoring system (https://qrisk.org/2017/).

### Assessment of Conventional Cardiovascular Risk Markers

Anthropometric data included height, weight and body mass index (BMI), the latter recorded using the Tanita MC-780MA P (Tanita Europe BV, Netherlands). Systolic blood pressure (SBP) and diastolic blood pressure (DBP) were recorded as the average of three using an Omron M2 Upper Arm Blood Pressure Monitor (OMRON Healthcare Europe BV, United Kingdom). An overnight fasting blood sample of approximately 9 ml was taken into a serum-separating tube (Greiner Bio-One, Gloucestershire, United Kingdom) for the analysis of blood lipid and glucose concentrations. Serum-separating tubes were kept upright at room temperature for 30 min (and no more than 60 min) before centrifuging at 1,700 *xg* for 15 min at room temperature. The plasma (160 μl) was collected and analyzed by iLab (iLab 600 Clinical Chemistry System, Diamond Diagnostics, United States) for triacylglycerol (TAG), total cholesterol (TC), high-density lipoprotein cholesterol (HDL-C), and glucose concentrations using standard reagent kits (Werfen Limited, Warrington, United Kingdom).

### Blood Collection and Processing

Venous blood samples were collected from fasted subjects into vacutainer tubes containing 3.2% sodium citrate (Greiner Bio-One, Gloucestershire, United Kingdom), and were kept at room temperature and processed within 30 min after transferring into polypropylene tubes (Fisher Scientific, Loughborough, United Kingdom). Platelet-rich plasma (PRP) was prepared by centrifugation of whole blood at 175 *xg* for 15 min, with no brake at room temperature. Platelet-poor plasma (PPP) was obtained by centrifugation of PRP at 13,000 *xg* at room temperature for 2 min and removal of the supernatant from the platelet pellet. To obtain platelet-free plasma (PFP), whole blood was centrifuged at 1,500 *xg* for 15 min at room temperature and the upper two-thirds were collected and centrifuged once again at 13,000 *xg* for 2 min at room temperature, before collecting the upper three quarters from each tube as PFP. Freshly prepared PRP and PPP were used in the platelet aggregation assay. Some of the PFP was used for the enumeration and characterization of circulating EVs, while the remainder was stored at −80 °C for the analysis of thrombogenic activity of EVs (thrombin generation) and of PFP (thrombin generation and fibrin clot properties).

### Isolation, Enumeration and Characterization of Circulating EVs

Fresh PFP (500 μl) was subjected to size exclusion chromatography (SEC) using Izon qEV columns (Izon Science Ltd, Oxford, United Kingdom) to isolate EVs prior to nanoparticle tracking analysis (NTA). Phosphate-buffered saline (PBS) was used to elute 10 sequential fractions of 0.5 ml and fractions 7–9 were diluted with PBS to achieve the recommended concentration range of particles (1–10 × 10^8^ vesicles/ml) (26, 27) for the analysis on a NanoSight 300 (Malvern, Amesbury, United Kingdom). Data from five separate videos, each of 60 s' duration, were analyzed using the NTA 3.20 instrument software, which identifies individual particles and estimates size based on the Stokes-Einstein Equation (28). A threshold of 70 nm was set to ensure minimal interference by lipoproteins. EVs enumerated by NTA in this way were defined as total EVs (TEVs).

### Enumeration and Characterization of Circulating EVs Using Flow Cytometry (FCM)

For enumeration and phenotyping of circulating EVs by FCM (BD FACSCanto II flow cytometer, BD Biosciences, Wokingham, United Kingdom), an initial EV size gate was set using non-fluorescent, silica ApogeeMix beads with diameters of 180 nm, 240 nm, 300 nm, 590 nm, 880 nm, and 1,300 nm (Apogee flow systems, Hemel Hempstead, United Kingdom) and additionally, fluorescent latex beads with diameters of 110 nm and 500 nm. The 240 nm silica beads corresponded to the lowest reliable detection limit of the flow cytometer and any particles below this size (on side scatter mode) were excluded to minimize background noise. In order to exclude platelets and cellular material, the upper detection gate would ideally be set at 1 μm, but the closest available diameter of silica beads was 880 nm, so the gate was set just above this on scatter mode.

The following fluorochrome-coupled antibodies and their corresponding isotypes were used for EV phenotyping: Annexin V conjugated to Allophycocyannin (APC) (Thermo Fisher Scientific, Basingstoke, United Kingdom) for phosphatidylserine (PS) positive EVs, anti-CD41 conjugated to Phycoerythrin (PE) (Diagnostica Stago LTD, Theale, United Kingdom) for platelet-derived EVs (PDEVs) and anti-CD105 conjugated to eFluor450 (Life Technologies LTD (Invitrogen division), Paisley, United Kingdom) for endothelial-derived EVs (EDEVs). In order to minimize the background caused by nonspecific antibody binding, a panel of negative controls were included in the analysis. For Annexin V labeling, the negative control was PFP with Annexin V-APC, but Annexin V did not bind to PS due to the absence of Ca+; negative controls for CD41 and CD105 were PFP staining with the matching isotype controls (IgG1-PE and IgG1-eFlour450, respectively). Fresh PFP (5 μl) was incubated with FcR blocking reagent (5 μl) (Miltenyi Biotec Ltd, Surrey, United Kingdom) and Annexin V buffer (10 mM HEPES, 140 mM NaCl, 2.5 mM CaCl2, pH 7.40; Cambridge Bioscience, Cambridge, United Kingdom) for 15 min in the dark at room temperature, before the incubation with antibodies or isotype-matched controls for a further 15 min in the dark at room temperature. Samples were then diluted with 200 μl Annexin V buffer and analyzed by FCM. EVs staining positive for Annexin V were classified as PS+EVs, and the final numbers of PS+EVs were obtained by Annexin V+ events recorded in the main sample tube subtracting events recorded in the Annexin V control tube; those staining positive for both Annexin V and CD41 were classified as PDEVs, in which a cut-off as CD41+ particles to 1% was set in events recorded in the CD41 isotype control tube and then the final numbers of PDEVs were obtained from the events recorded in the main sample tube; those staining positive for both Annexin V and CD105 were classified as EDEVs, in which a cut-off as CD105+ particles to 1% was set in events recorded in the CD105 isotype control tube and then the final numbers of EDEVs were obtained from the events recorded in the main sample tube (Supplementary Figures 1A–F). Absolute numbers of vesicles were calculated using BD Trucount tubes (BD Biosciences, Wokingham, United Kingdom).

### Assessment of Thrombin Generation Triggered by Circulating EVs

Thawed PFP (500 μl) was subjected to SEC for the isolation and purification of EVs as described above. Fractions 7–9 were pooled and concentrated using a centrifugal concentrator (Fisher Scientific, Leicestershire, United Kingdom) at 1000 *xg* for 10 min at room temperature. The protein concentration of EV suspensions was assessed using a Nanodrop-1000 spectrophotometer (Thermo Scientific, Loughborough, United Kingdom) and adjusted to 50 μg/ml in at least 25 μl. A stock of vesicle-free plasma (VFP) from healthy volunteers was prepared (29) and stored in aliquots at −80 °C prior to use as a reference against which EV-specific thrombin generation was assessed. Thrombin generation was assessed using a commercially available thrombin generation assay, based on the cleavage of a fluorogenic substrate by thrombin upon activation of the clotting cascade, according to the manufacturer's instructions (Technothrombin TGA kit, Austria). Briefly, a 10 μl EV suspension at a concentration of 50 μg protein/ml or PBS (as a control) was added to a 30 μl of VFP stock, followed by the addition of 10 μl of a low concentration of phospholipid micelles containing recombinant human tissue factor (TF) in Tris-Hepes-NaCl buffer. The plate was then immediately read at 37 °C for 1 h at 1-min intervals using a FlexStation 3 Multi-Mode microplate reader (Molecular Devices Limited, Wokingham, United Kingdom) at 360 nm for excitation and 460 nm for emission. All samples were analyzed in duplicate and data were analyzed by the TECHNOTHROMBIN® TGA Evaluation Software to present five variables: lag time (min), time to the peak (min), peak concentration of thrombin (nM), velocity-index (nM thrombin/min) and area under curve (defined as endogenous thrombin potential (ETP), nM thrombin × min). The thrombogenicity of circulating EVs was expressed as the capacity to elicit TF-dependent thrombin generation and was calculated as thrombin generation resulting from purified circulating EVs in VFP minus that resulting from VFP alone.

### Assessment of Thrombin Generation in PFP

A 40 μl aliquot of thawed PFP was added into 10 μl of a low concentration of phospholipid micelles in the thrombin generation assay and overall thrombin generation was assessed described above.

### Assessment of Clot Growth and Fibrinolysis in PFP

Clot growth and fibrinolysis in samples of PFP were assessed using a thrombodynamics analyzer and thrombodynamics kit (HemaCore, Moscow, Russia). PFP (120 μl) was thawed in a 37 °C water bath for 5 min. Plasma was placed into a plastic vial containing a lyophilized solution of corn trypsin inhibitor to prevent contact activation, followed by incubation with 4 nM of tissue-type plasminogen activator (Sigma-Aldrich, Dorset, United Kingdom) for 15 min at 37 °C within the thermostat of the analyzer and then the addition of a lyophilized solution of calcium salt. The sample was immediately transferred into an optically transparent cuvette with two thin channels, which were placed into the 37 °C temperature-controlled chambers of the instrument. Finally, an activating insert, the end edges of which were covered with immobilized TF to activate clotting, was gently placed fully into the cuvette. Growth and lysis of the fibrin clot were quantified using video microscopy software over a 60 min period (see Supplementary Figures 2A–C).

### Assessment of Platelet Aggregation

Freshly prepared PRP (40 μl) was added to appropriate wells of a microplate containing five separate agonists: adenosine diphosphate (ADP), cross-linked collagen-related peptide (CRP-XL), epinephrine, thrombin receptor-activated peptide 6 (TRAP-6) and U46619, and the plate was tapped gently to ensure that PRP reached the bottom. The plate was shaken at 1,200 *rpm* at 37 °C for 5 minutes using a plate shaker (Thermo-shaker, Grant Instruments, United Kingdom). PRP or PPP were also added to control wells containing no agonist. A plate-reader (Tecan Microplate Reader Spark, Switzerland) was used to determine the absorbance at 405 nm. Absorbance data were converted to percent aggregation by reference to the absorbance for the PRP control being 0% and that for PPP being 100%. Dose-response curves for each agonist were obtained and curves were fitted by a four-parameter logistic non-linear regression using Prism (Version 8.2, GraphPad Software, Inc., California).

## Statistical Analysis

Data are expressed as mean ± SEM if normally distributed and as median with inter-quartile range when not. Data were analyzed for normality and equal variance, and all variables with non-normal distribution were log-transformed to achieve normal distribution before correlation analysis. The strengths of the correlations between EV parameters and risk parameters were calculated by Pearson's correlation coefficient. Significantly associated variables were first entered into a univariate regression model separately and then into a multivariate regression model to check the interference of each associated variable on the explanation of EV parameters. In order to identify independent predictors of EV numbers, all variables with *p*-values < 0.05 in the univariate regression model were incorporated into a stepwise multivariate regression model, in which parameters of F ≤ 0.05 were entered and F ≥ 0.10 were removed. Same regression analysis were also constructed to identify the roles of EV parameters and conventional risk markers in the explanation of thrombogenic risk markers. The association between the quartile range of EV numbers and 10-year CVD risk detected by QRISK2 was examined by analysis of covariance (ANCOVA) followed by the Bonferroni post hoc test. All statistical analyses were performed with SPSS Statistics version 25 and a *p*-value < 0.05 was considered statistically significant.

## Results

### Characteristics of the Study Population

Subject characteristics are shown in Table 1 and Supplementary Table 1. The median age was 65 years, and 24 out of 40 subjects were male. The mean 10-year CVD risk score was 12.9%, which was at the lower end of the QRISK2 definition of moderate risk of CVDs (10–20%). The mean diameter of circulating EVs was 98.0 nm and 96% of TEVs detected by NTA had a diameter < 200 nm.

**Table 1:** Subjects characteristics.

**Sample characteristics**	**All subjects (*n* = 40)**
Age (years)	65 (5)
Male: Female ratio	24:16
BMI (kg/m^2^)	25.4 ± 0.5
SBP (mmHg)	134 ± 2.2
DBP(mmHg)	79 ± 1.4
TC (mmol/L)	6.0 ± 0.2
TAG (mmol/L)	1.3 ± 0.1
HDL-C(mmol/L)	1.6(0.3)
TC/HDL-C ratio	3.9 ± 0.1
Glucose (mmol/L)	5.8 ± 0.1
10-year CVD risk score (%) (QRISK2)	12.9 ± 0.01

*Normally distributed data are shown as mean ± SEM and non-normally distributed data as median (inter-quartile range). BMI, body mass index; BP, blood pressure; CVD, cardiovascular disease; DBP, diastolic blood pressure; HDL-C, high-density lipoprotein cholesterol; SBP, systolic blood pressure; TAG, triacylglycerol; TC, total cholesterol*.

### BMI, BP and Plasma TAG Concentration Are Positively Associated With Circulating EV Numbers

**Figure 1:**
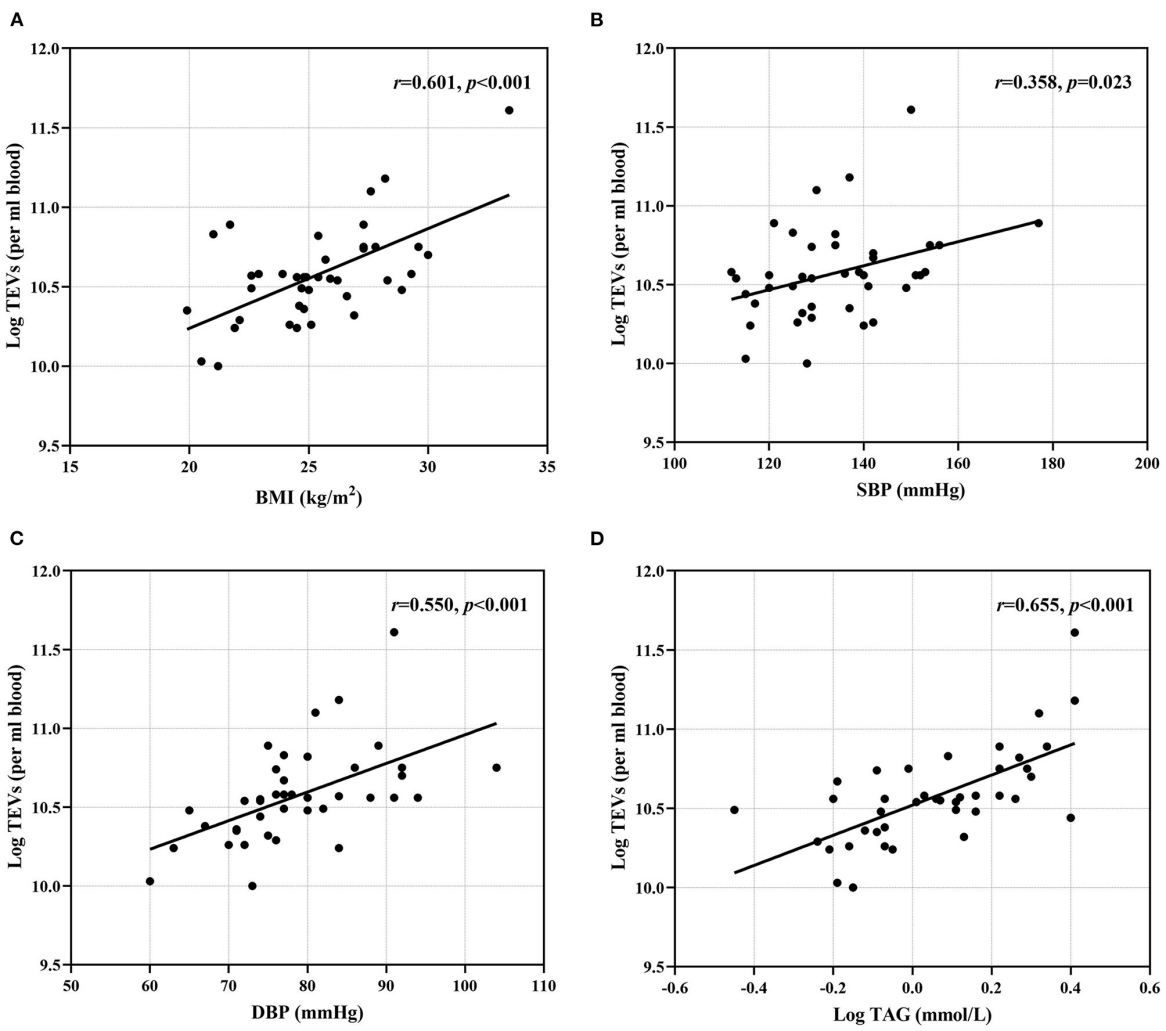
Associations between conventional risk markers and EV numbers**. (A)** BMI; **(B)** SBP; **(C)** DBP and **(D)** plasma TAG were positively correlated with TEV numbers. BMI, body mass index; DBP, diastolic blood pressure; SBP, systolic blood pressure; TAG, triacylglycerol; TEVs, total extracellular vesicles.

BMI, SBP, DBP and plasma TAG concentration were positively associated with numbers of circulating TEVs (Figures 1A–D and Supplementary Table 2). Plasma TC concentration was positively correlated with numbers of PDEVs (*r* =0.330, *p* = 0.038), but there were no other associations of any risk markers with numbers of PS+EVs, PDEVs or EDEVs (Supplementary Table 2). The mean size of EVs overall, as assessed by NTA, was inversely associated with BMI (*r* = −0.316, *p* = 0.047) and plasma TAG concentration (*r* = −0.395, *p* = 0.012) (Supplementary Table 2).

### Circulating EVs Numbers Are Associated With Some Aspects of Thrombogenic Activity *ex vivo*

Higher numbers of TEVs, as determined by NTA, were associated with increased thrombin generation, reflected by the lag time for thrombin generation (*r* = −0.382, *p* = 0.015), peak thrombin concentration (*r* = 0.588, *p* < 0.001), time to peak thrombin concentration (*r* = −0.453, *p* = 0.003), velocity index (*r* = 0.578, *p* < 0.001) and ETP (*r* = 0.563, *p* < 0.001), while the numbers of PS+EVs, PDEVs and EDEVs were not associated with thrombin generation (Supplementary Table 3). Higher numbers of TEVs were associated with enhanced clot formation, reflected by the rate of clot growth (*r* = 0.569, *p* < 0.001) and clot size at 30 min (*r* = 0.481, *p* = 0.002), but numbers of PS+EVs, PDEVs and EDEVs were not associated with any parameters of clot formation (Supplementary Table 3). Higher numbers of EDEVs were related to a higher tendency for clot lysis, indicated by an inverse relationship with lysis onset time (*r* = −0.420, *p* = 0.007) and a positive association with lysis progression (*r* = 0.469, *p* = 0.002) (Supplementary Table 3). However, EV numbers did not relate to platelet aggregation induced by ADP, CRP-XL, epinephrine, TRAP-6 or U46619 (Supplementary Table 3). There was no relationship between EV size and thrombin generation, fibrin clot properties or platelet aggregation, apart from an inverse association between the mean size of EVs and the initial rate of clot growth (*r* = −0.330, *p* = 0.038), a positive correlation between the mean size of EVs and epinephrine-induced platelet aggregation (*r* = 0.314, *p* = 0.048) and a negative correlation between the mean and mode size of EVs with U46619-induced platelet aggregation (*r* = −0.338, *p* = 0.033; *r* = −0.382, *p* = 0.015, respectively) (Supplementary Table 3).

### Regression Analysis Supports Strong Relationships Between EV Numbers, Plasma TAG Concentration and Thrombogenic Markers

In a multivariate regression model, plasma TAG concentration emerged as the strongest CVD risk marker to be associated with numbers of circulating EVs, independently predicting numbers of circulating EVs (Table 2). Stepwise regression analysis demonstrated that plasma TAG concentration explained 49% of the variance for TEV numbers and an additional 9% of the variance in TEV numbers was accounted for by DBP (Table 3).

**Table 2:** Associations between TEV numbers and conventional risk markers.

	**TEVs (per ml blood)**			
	**Univariate analysis**		**Multivariate analysis** **R**^**2**^ **=** **0.630** ***p*** **<** **0.001**	
	**b**	***p***	**b**	***p***
BMI (kg/m^2^)	0.063	<0.001	0.023	0.096
SBP (mmHg)	0.008	0.023	0.003	0.367
DBP (mmHg)	0.018	<0.001	0.005	0.370
TAG (mmol/L)	0.358	<0.001	0.261	<0.001

*In univariate analysis, BMI, SBP, DBP and TAG concentration were significant factors for TEV numbers, while only plasma TAG concentration was still significantly correlated with TEV when entered into a multivariate model (R^2^ = 0.630, p < 0.001). BMI, body mass index; DBP, diastolic blood pressure; SBP, systolic blood pressure; TAG, triacylglycerol; TEVs, total extracellular vesicles*.

**Table 3:** Independent predictors of TEV numbers determined by stepwise regression.

	**TEVs (per ml blood)**				
	**Model**	**b** **(95% CI)**	**SE (b)**	**β**	***p*** **-value**
1	(Constant)	10.120 (9.954, 10.286)	0.082		<0.001
	TAG R^2^ = 0.494	0.358 (0.239, 0.477)	0.059	0.703	<0.001
2	(Constant)	9.336 (8.767, 9.906)	0.281		<0.001
	TAG	0.294 (0.176, 0.411)	0.058	0.577	<0.001
	DBP	0.011 (0.003, 0.019)	0.004	0.330	0.006
	R^2^ = 0.588				

*Unstandardized coefficients (b) indicates that as the independent variables (TAG and DBP) change by one unit, the dependent variable (TEV numbers) change by b units. Regression coefficient (β) indicates that as the independent variables change by 1 SD, the dependent variable change by β SD. For reference, 1-SD of TAG concentration is 0.6 mmol/L; 1-SD of DBP level is 9.1 mmHg; 1-SD of log TEV numbers is 0.30/ log ml blood. CI, confidence interval; DBP, diastolic blood pressure; SD, standard deviation; SE, standard error; TAG, triacylglycerol; TEVs, total extracellular vesicles*.

Besides TEV numbers, BMI, DBP and TAG were also significantly associated with increased thrombin generation as univariate determinants, reflected by the lag time for thrombin generation, peak thrombin concentration, velocity index and ETP (data not shown); however, only TEV numbers remained significantly associated with thrombin generation when they were entered into a multivariate regression model (Table 4). Stepwise regression analysis suggested that TEV numbers explained 15% of the variance for lag time, 35% of the variance for peak thrombin concentration, 33% of the variance for velocity index and 32% of the variance for ETP, respectively (Table 5). DBP, TAG and HDL-C were also significantly associated with increased clot formation, reflected by the rate of clot growth and the clot size at 30 min (data not shown). When univariate determinants of parameters were entered into a multivariate regression model, TEV numbers remained significantly associated with the rate of clot growth and HDL-C concentration remained significantly associated with clot size at 30 min, respectively (Table 4). Finally, stepwise regression analysis suggested that circulating TEV numbers explained 32% of the variance for the rate of clot growth and 13% of the variance for clot size at 30 min. An additional 23% of the variance in clot size at 30 min was predicted by HDL-C concentration (Table 5).

**Table 4:** Association of thrombogenic risk markers with EV numbers and conventional risk markers.

	**Lag time** **(min)**				**Peak thrombin concentration** **(nM)**				**Velocity index** **(nM thrombin/min)**				**ETP** **(nM thrombin** **× min)**			
	**Univariate analysis**		**Multivariate analysis** **R**^**2**^ **=** **0.169** ***p*** **=** **0.081**		**Univariate analysis**		**Multivariate analysis** **R**^**2**^ **=** **0.355** ***p*** **=** **0.001**		**Univariate analysis**		**Multivariate analysis** **R**^**2**^ **=** **0.337** ***p*** **=** **0.005**		**Univariate analysis**		**Multivariate analysis** **R**^**2**^ **=** **0.323** ***p*** **=** **0.003**	
	**b**	***p***	**b**	***p***	**b**	***p***	**b**	***p***	**b**	***p***	**b**	***p***	**b**	***p***	**b**	***p***
**BMI** **(kg/m**^**2**^**)**	−0.377	0.048	−0.129	0.583	0.023	0.005	0.007	0.476	0.029	0.018	0.004	0.796	0.030	0.011	0.007	0.574
**DBP** **(mmHg)**	–	**–**	–	–	–	**–**	–	–	0.008	0.038	0.000	0.963	–	**–**	–	–
**TAG** **(mmol/L)**	−5.800	0.029	−2.427	0.485	0.305	0.010	0.009	0.950	0.382	0.026	−0.060	0.768	0.379	0.021	−0.027	0.890
**TEVs** **(per ml blood)**	−4.401	0.015	−2.572	0.319	0.303	<0.001	0.261	0.013	0.429	<0.001	0.432	0.011	0.400	<0.001	0.370	0.013
	**Rate of clot growth (μm/min)**				**Clot size (μm)**			
	**Univariate analysis**		**Multivariate analysis** **R**^**2**^ **=** **0.334** ***p*** **=** **0.002**		**Univariate analysis**		**Multivariate analysis** **R**^**2**^ **=** **0.378** ***p*** **<** **0.001**	
	**b**	***p***	**b**	***p***	**b**	***p***	**b**	***p***
DBP (mmHg)	0.001	0.026	0.000	0.718	–	**–**	–	–
TAG (mmol/L)	0.066	0.004	0.018	0.505	0.063	0.005	0.021	0.411
HDL (mmol/L)	–	–	–	–	−0.196	0.002	−0.148	0.012
TEVs (per ml blood)	0.059	<0.001	0.047	0.030	0.047	0.002	0.028	0.109

*In univariate analysis, TEV numbers was significant factor for lag time, peak thrombin concentration, velocity index and ETP; BMI and TAG were significant factors for all thrombin generation parameters and DBP was significant factor for velocity index; however, only TEV numbers was still significantly correlated with thrombin generation-related parameters when entered into multivariate model. TEV numbers, DBP and TAG concentration were significant factors for the rate of clot growth, while TEV numbers, plasma TAG and HDL concentrations were significant factors for clot size at 30 min. Only TEV numbers was still significantly correlated with the rate of clot growth, as well as plasma HDL concentration being still significantly correlated with clot size at 30 min when entered into multivariate model. BMI, body mass index; DBP, diastolic blood pressure; HDL-C, high-density lipoprotein cholesterol; TAG, triacylglycerol; TEVs, total extracellular vesicles.*

**Table 5:** Independent predictors of thrombogenic risk markers determined by stepwise regression.

		**Model**	**b (95% CI)**	**SE (b)**	**β**	**p–value**
**Lag time**	**1**	(Constant)	73.574 (36.612, 110.536)	18.258		<0.001
		TEV numbers R^2^ = 0.146	−4.401 (−7.895, −0.907)	1.726	−0.382	0.015
**Peak thrombin concentration**	**1**	(Constant)	−1.337 (−2.787, 0.113)	0.716		0.070
		TEV numbers R^2^ = 0.345	0.303 (0.166, 0.440)	0.068	0.588	<0.001
**Velocity index**	1	(Constant)	−3.975 (−6.080, −1.870)	1.040		<0.001
		TEV numbers R^2^ = 0.334	0.429 (0.230, 0.628)	0.098	0.578	<0.001
**ETP**	1	(Constant)	−1.069 (−3.111, 0.973)	1.009		0.296
		TEV numbers R^2^ = 0.317	0.400 (0.207, 0.593)	0.095	0.563	<0.001
**Rate of clot growth**	1	(Constant)	0.891 (0.596, 1.186)	0.146		<0.001
		TEV numbers R^2^ = 0.324	0.059 (0.031, 0.087)	0.014	0.569	<0.001
**Clot size** **at 30 min**	**1**	(Constant)	3.155 (3.131, 3.179)	0.012		<0.001
		HDL R^2^ = 0.233	−0.196 (−0.314, −0.079)	0.058	−0.482	0.002
	**2**	(Constant)	2.753 (2.460, 3.046)	0.145		<0.001
		HDL	−0.155 (−0.267, −0.043)	0.055	−0.381	0.008
		TEV numbers R^2^ = 0.366	0.037 (0.010, 0.064)	0.013	0.379	0.008

*Unstandardized coefficients (b) indicates that as the independent variables (TEV numbers and/or HDL concentration) change by one unit, the dependent variable (thrombin generation parameters: lag time for thrombin generation, peak thrombin concentration, velocity index and ETP; clot formation parameters: rate of clot growth and clot size at 30 min) change by b units. Regression coefficient (β) indicates that as the independent variables change by 1 SD, the dependent variable change by β SD. For reference, For reference, 1-SD of log TEV numbers is 0.30/ log ml blood; 1-SD of log HDL is 0.24 log mmol/L; 1-SD of log lag time is 0.06 log min; 1-SD of log the level of peak thrombin concentration is 0.16 log nM; 1-SD of log velocity index is 0.23 log nM thrombin/min; 1-SD of log ETP is 0.22 nM thrombin × min; 1-SD of log rate of clot growth is 0.03 log μm/min; 1-SD of log clot size at 30 min is 0.03 log μm; CI, confidence interval; ETP, endogenous thrombin potential; HDL-C, high-density lipoprotein cholesterol; SD, standard deviation; SE, standard error; TEVs, total extracellular vesicles*.

### EVs Isolated From Subjects With Moderate CVD Risk Increase TF-Dependent Thrombin Generation *ex vivo*

**Figure 2:**
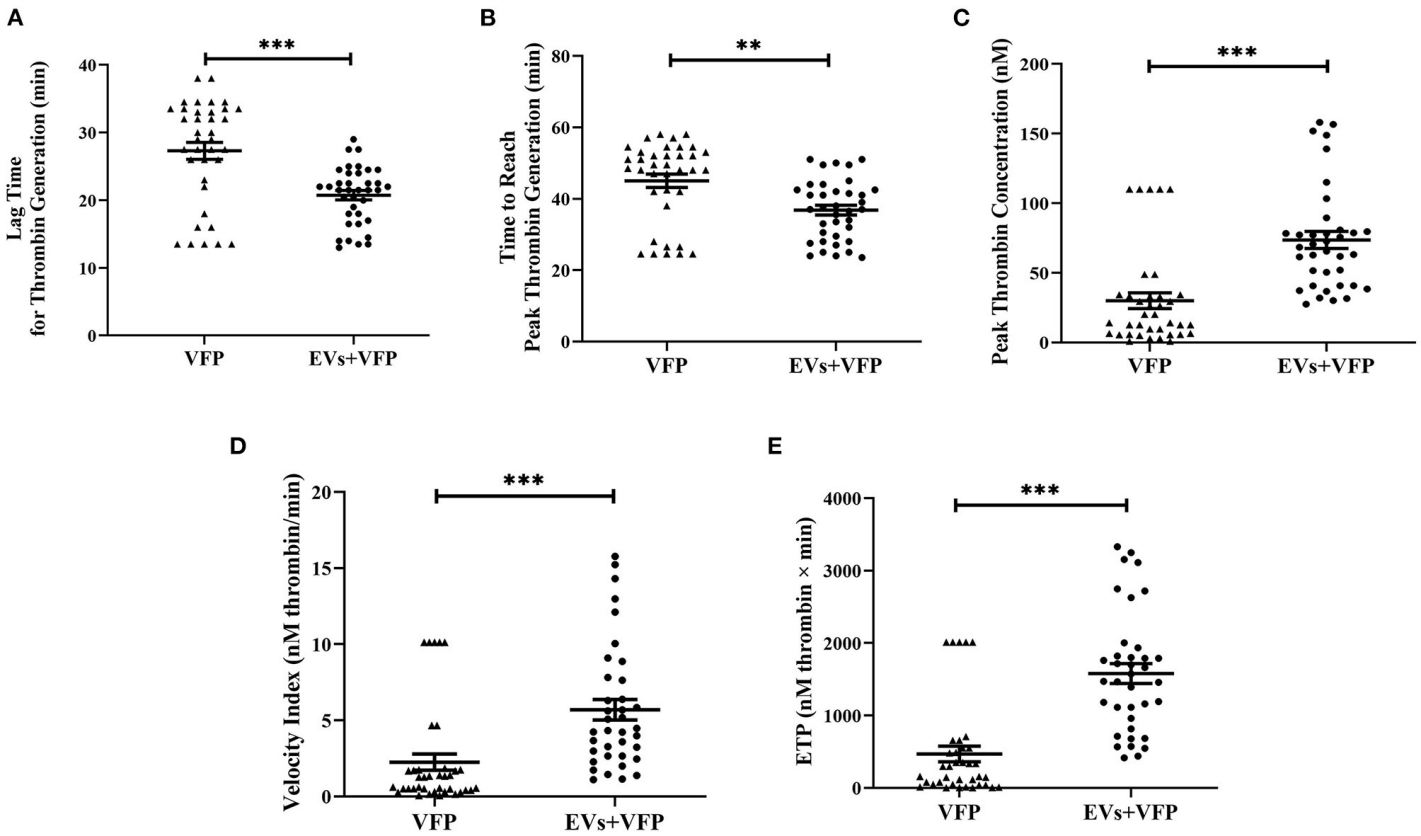
Thrombogenicity of circulating EVs in activating TF-dependent thrombin generation. **(A)** Data are mean ± SEM, triangles represent the TF-dependent thrombin generation exhibited in VFP of each individual and dots represent the TF-dependent thrombin generation exhibited in VFP with the addition of circulating EVs of each individual. Addition of circulating EVs into VFP significantly shortened the lag time for thrombin generation and **(B)** the time to reach peak thrombin generation **(C)** significantly increased peak thrombin concentration; **(D)** velocity index and **(E)** ETP compared to VFP alone. **p < 0.01 and ***p < 0.001. ETP, endogenous thrombin potential; EVs, extracellular vesicles; VFP, vesicle-free plasma.

Addition of circulating EVs to VFP significantly shortened the lag time for thrombin generation (*p* = 0.001) and the time to reach peak thrombin generation (*p* = 0.002), as well as significantly increasing peak thrombin concentration (*p* < 0.001), velocity index (*p* < 0.001) and ETP (*p* < 0.001) compared to VFP alone, demonstrating that TF-dependent thrombin generation was promoted by circulating EVs (Figures 2A–E). The mode size of EVs was inversely associated with peak thrombin concentration (*r* = −0.358, *p* = 0.025), velocity index (*r* = −0.366, *p* = 0.022) and ETP (*r* = −0.413, *p* = 0.009), and positively associated with the time to reach peak thrombin concentration (*r* = 0.335, *p* = 0.037), suggesting that smaller EVs had a greater capacity to promote thrombin-generation (data not shown).

### Positive Association Between Circulating EV Numbers and 10-Year CVD Risk Score

**Figure 3:**
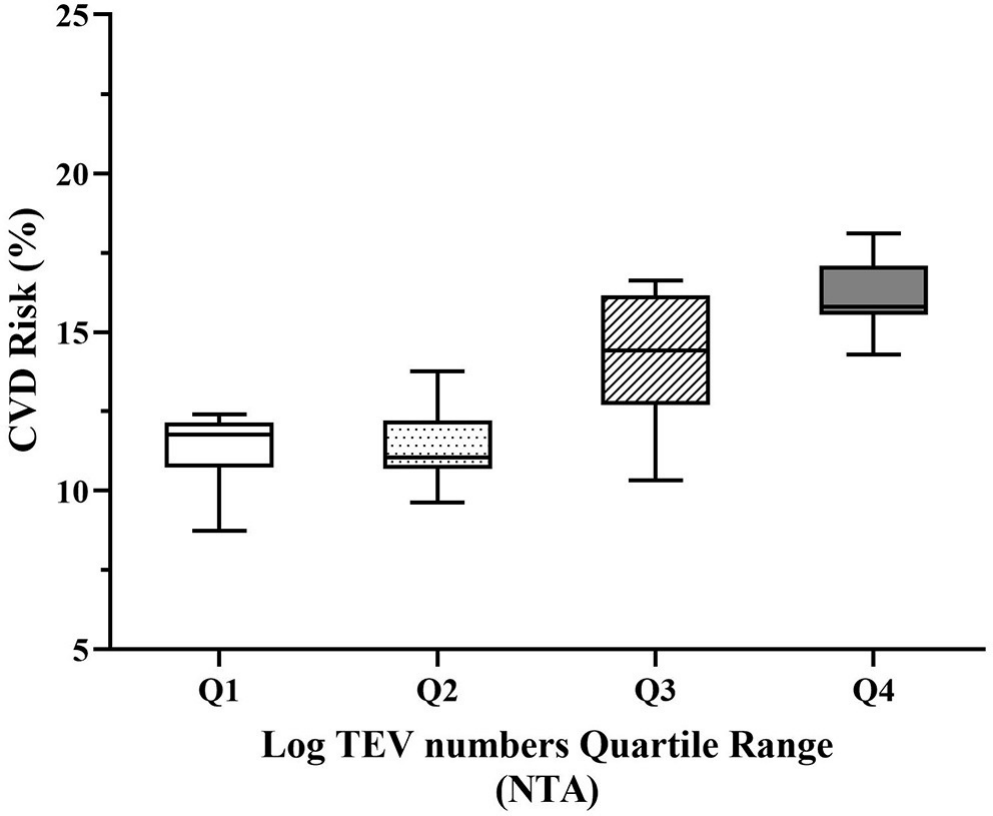
Adjusted 10-year CVD risk relative to quartiles of circulating EV numbers. Increasing quartiles of TEV numbers were associated with increased 10-year CVD risk adjusted for age, plasma TAG and HDL-C (*p* < 0.001). TEVs, TAG and HDL-C were log-transformed to achieve normal distribution before analysis. CVD, cardiovascular diseases; HDL-C, high-density lipoprotein cholesterol; NTA, nanoparticle tracking analysis; TAG, triacylglycerol; TEVs, total extracellular vesicles.

Subjects in the highest quartiles of TEV numbers had significantly higher 10-year CVD risk scores when evaluated (i) without adjustment, (ii) after adjusting for age only, (iii) after adjusting for age and plasma TAG concentration or (iv) after adjusting for age, plasma TAG and HDL-C concentrations, all of which were associated with 10-year CVD risk score and were therefore regarded as covariates (Figure 3; Supplementary Table 4).

## Discussion

This study demonstrates a strong relationship between numbers of circulating EVs and BMI, BP, plasma TAG concentration and thrombogenic markers. Plasma TAG concentration and DBP independently predicted numbers of circulating EVs, while the number of circulating EVs was an independent predictor of aspects of thrombin generation and clot formation (with the addition of plasma HDL-C concentration) in stepwise regression analysis. EVs isolated from subjects with moderate CVD risk promoted thrombin generation in VFP *ex vivo*, demonstrating a direct link between EVs and thrombogenic activity. Subjects in the highest quartiles of circulating EV numbers had significantly greater 10-year CVD risk, independent of other conventional cardiovascular risk markers, indicating that elevated numbers of EVs were independently associated with 10-year CVD risk.

The use of FCM for enumeration and characterization of EVs, while common, is limited in its ability to detect very small EVs due to its threshold ranging from about 200–500 nm and the lack of universal fluorescent marker for labeling all type of EVs (30, 31). Although most studies investigating circulating EVs consider that PS-exposing EVs labeled with Annexin V represent the majority of EVs, it is clear that not all circulating EVs are Annexin V positive (32, 33). NTA, on the other hand, can resolve smaller vesicles that cannot be detected by FCM to enable a more complete and accurate representation of the circulating EV population, although it cannot accurately quantify the smallest exosomes and lacks the ability to phenotype EVs. A combination of NTA and FCM therefore provides a fuller picture of the EV population, although techniques which provide a complete picture are still lacking.

There are previous reports of an association between circulating EV numbers and CVD risk markers, although most studies have used FCM rather than NTA to enumerate EVs. Two studies indicated a positive association between fasting plasma TAG concentrations of healthy subjects and TEV numbers assessed by NTA (34, 35). Amosse et al. further reported that EV numbers assessed by NTA were positively correlated with both BMI and plasma TAG concentration (22). Multivariate regression analysis in the current study demonstrated that TAG and DBP are independent predictors for TEV. The association between plasma TAG concentration and risk of CVDs is well-established, although there is still some disagreement about whether plasma TAG concentration is an independent risk marker of CVDs, as its impact may be attenuated when cholesterol and lipoprotein metabolism are taken into account (36, 37). In the current study and in the analysis by Amabile et al., the relationship between plasma TAG concentration and TEV/EDEV numbers was independent of TC and HDL-C concentrations (38). Ferreira et al. also showed that EDEV numbers were elevated after a high-fat meal, accompanying the postprandial increase in plasma TAG concentration (25). Furthermore, SBP predicts cardiovascular morbidity and mortality, particularly above 50 years of age, and to a greater degree than DBP (39). In the current study, where the mean age of the subjects was 65 years, DBP rather than SBP independently predicted EV numbers. Although the mechanism of EV generation is still under debate, EV release can be promoted by endothelial dysfunction, platelet activation and inflammation, all of which are influenced by these conventional risk factors (14, 16). For example, impaired nitric oxide bioavailability and/or signaling have been observed in obesity (40), hypertension (41) and hypertriglyceridemia (42), and is one of the main characteristics of endothelial dysfunction. Platelet activation can be also promoted by increased shear force due to hypertension (43) or by very low density lipoprotein cholesterol accumulation in hypertriglyceridemia (44). Excessive adipose tissue exhibited in overweight or obesity (45) and increased BP in hypertensive patients (46) are associated with the elevated production of proinflammatory cytokines, including tumor necrosis factor-α (TNF-α), interleukin-1 β (IL)-1β, and IL-6, and these cytokines can enhance PDEV generation *in vivo* in the presence of high shear stress and platelet activation (47) or trigger the release of EDEVs from human umbilical vein endothelial cells (48), pulmonary microvascular and aortic endothelial cells *ex vivo* (49), due to their effects on vesiculation of the endothelial plasma membrane.

In the current study, the addition of circulating EVs isolated from subjects with moderate risk of CVDs to VFP promoted TF-dependent thrombin generation, which is consistent with previous studies suggesting that EVs have thrombogenic capability (50, 51). Also, to the best of our knowledge, this is the first study demonstrating that numbers of circulating EVs are associated with a range of *ex vivo* thrombogenic markers in subjects with moderate risk of CVDs, including thrombin generation parameters, the rate of clot growth, clot size and clot lysis. The procoagulatory properties of EVs may be due to the presence of anionic phospholipids, particularly PS, and the expression of TF on the surface of EVs. The externalized negative phospholipids on the surface of EVs provide a surface for the binding of coagulation Factors (F)VII, FIX and FX, and favor the assembly of coagulation complexes, contributing to thrombin generation and clot growth (29, 52). The procoagulatory ability of EVs is further enhanced by the expression of TF, which binds with FVIIa, activating FX and initiating the extrinsic pathway of the coagulation cascade, which converts fibrinogen to fibrin (53, 54). Other procoagulant molecules and receptors, such as P-selectin glycoprotein ligand-1 and CD42b, can be carried by EVs, enabling them to interact with CD62P and von Willebrand factor expressed on activated platelets and endothelial cells, respectively, contributing to thrombus propagation as well (18, 55, 56). An indirect role for EVs in thrombus formation was demonstrated by Suades et al. (57), who reported that the addition of EVs isolated from healthy subjects increased platelet deposition in damaged arteries *ex vivo*. Similarly, the expression and activity of TF on the surface of human umbilical vein endothelial cells were enhanced due to the stimulation of monocyte-derived EVs (58). However, it was unexpected that neither numbers of TEVs nor EV subtypes were associated with platelet aggregation induced by different agonists in the current study, which is inconsistent with previous findings about the role of EVs in inducing platelet aggregation (57, 59, 60). It is important to note that in the current study, thrombin generation, clot growth and fibrinolysis were assessed in the absence of platelets in order to define the relationship between EV number and thrombogenic parameters in the same matrix, with only platelet aggregation being assessed in a more physiological matrix. Since platelets play a critical role in coagulation and since platelet activation and aggregation promote the release of EVs (14, 16), while EVs in turn promote platelet aggregation (59, 60), the observations presented in this paper would need to be replicated in platelet-rich plasma in order to fully draw out any clinical implications.

Studies employing FCM to characterize EV subtypes have generally demonstrated positive relationships between BMI, BP and plasma lipids and numbers of PS+EVs, PDEVs and EDEVs (21–24, 38, 61–64). However, in the current study, only plasma TC concentration was positively correlated with numbers of PDEVs, as demonstrated in three other studies (61, 63, 64). Depleting cholesterol from platelets with methyl-β-cyclodextrin renders them unable to produce PDEVs (65) and the membranes of PDEVs tend to have a greater cholesterol concentration than those of unstimulated platelets (66), suggesting that cholesterol is concentrated in PDEVs during their formation. It is not clear, however, whether plasma cholesterol concentration influences this in any way. Regarding the relationship between EV subtypes and thrombogenic markers, EDEVs detected by FCM were positively associated with clot lysis. Increased EDEV numbers have been reported in patients with acute coronary syndromes (67) and acute ischemic stroke (68), and EDEVs have been proposed to have potential roles in fibrinolytic activity, at least partly through the activation of plasminogen into plasmin on their surfaces (69, 70). The EV-induced thrombin generation is also likely to be an indirect mechanism for changes in fibrinolysis as thrombin formation can modulate clot stability by changing fibrin properties, including its network structure and resistance to fibrinolysis (71, 72). The lack of such an association for PS+EVs and PS+PDEVs might be considered surprising given that PDEVs represent the most abundant type of circulating EVs and are the major source of PS+EVs in blood (73, 74). Indeed, some studies have demonstrated an association of PS+EV and PDEV numbers with thrombin generation and/or clotting time (75, 76). However, it cannot be assumed that the majority of PDEVs express PS (32, 33) and in the absence of a universal EV marker, this study was limited to evaluating PS+PDEVs only. Expression of TF also varies amongst EVs derived from different cell types and it has been reported that EDEVs and leukocyte-derived EVs, but not PDEVs, support plasmin generation, suggesting that coagulatory activity may differ considerably for different EV subtypes (29, 52, 53). The finding that there were no other associations of risk markers with numbers of EV subtypes may also reflect the fact that the majority of previous studies were conducted in patients diagnosed with various diseases and therefore the EV profile may be substantially different. Also, as noted above, FCM reports on a minor fraction of the total EV population compared to NTA (26, 33, 35), so it is perhaps not surprising that TEVs assessed by NTA were more strongly associated with risk markers than EV subtypes assessed by FCM.

Besides the stronger relationship between TEVs of which 96% smaller than 200 nm and risk markers, the mean/mode size of EVs was inversely associated with BMI, plasma TAG concentration, the initial rate of clot growth, U46619-induced platelet aggregation and EV-induced thrombin generation in the current study, indicating that smaller EVs (i.e. exosomes) may play a more important role in CVD risk than larger EVs (i.e. microvesicles). Only one study separated small and large EVs from PFP from obese subjects with metabolic syndrome by differential centrifugations and attempted to compare the relationship between numbers of small and large EVs and cardiovascular risk markers using a combination of NTA and FCM. Partly in agreement with the current study, both small and large EVs were associated with increased CVD risk markers, although different isolation methods and detection thresholds were applied (22). Although both exosomes and microvesicles are small membrane-derived particles with similar structures, they differ in size, density and biogenesis, and they have specific signatures in composition, cellular origin and functional properties (14, 16). Cell culture-based and animal-based studies have compared the differences in the secretion and activity of exosomes and microvesicles. Durcin et al. reported that 3T3-L1 adipocytes secreted about 100-fold more exosomes than microvesicles, and they presented different lipid and protein characteristics, with microvesicles expressing more PS (77). Another study demonstrated that exosomes, but not microvesicles, from rats with pulmonary arterial hypertension modulated pulmonary vascular responses and induced the development of pulmonary arterial hypertension when they were injected into healthy rats (78). However, other studies found that microvesicles from several cancer cell lines possessed more procoagulant activity than exosomes (79) and platelet-derived and monocyte-derived exosomes did not induce thrombin generation (29). There are clearly a number of outstanding questions regarding the influence of EV size on their activities and the potentially different roles of exosomes and microvesicles in CVDs.

Overall, higher TEV numbers were associated with higher 10-year CVD risk score, both with and without adjustment for covariates, including age, TAG and HDL-C, but numbers of PS+EVs, PDEVs and EDEVs were not associated with 10-year CVD risk. Some previous studies have demonstrated no relationship (80), while others have demonstrated greater numbers of CD31+/CD41-EDEVs related with higher 10-year CVD risk using the Framingham Risk Score (38, 64) and higher PS+EV or EDEV numbers associated with major adverse cardiovascular events and death (19, 81). The current study used the QRISK2 score, which incorporates important factors such as ethnicity, clinical diseases and treatment information, and is regarded to perform well for discriminating different levels of risk in a United Kingdom population compared to the Framingham Risk Score (82, 83). Although a causal relationship is far from clear, the underlying mechanisms for EVs to play a role in the pathogenesis of CVD could include their procoagulatory properties, as well as other atherogenic pathways, such as endothelial dysfunction and inflammation (13, 18).

## Conclusion

In conclusion, this study demonstrated that circulating EVs were strongly associated with both conventional and thrombogenic risk markers of CVDs and overall CVD risk in subjects with moderate risk of CVDs, highlighting the potential for EVs as a novel predictive biomarker. Further studies exploring the potential mechanisms linking BP and plasma TAG concentration to the release of circulating EVs and the contribution of EVs to thrombogenic activity and CVDs are warranted.

## Data Availability Statement

The datasets presented in this study can be found in an online repository, which is the University of Reading Research Data Archive at https://doi.org/10.17864/1947.000366.

## Ethics Statement

The studies involving human participants were reviewed and approved by University of Reading Research Ethics Committee (UREC 17/18). The patients/participants provided their written informed consent to participate in this study.

## Author Contributions

PY designed the study and RZ and EB conducted the study, analyzed the samples and conducted the statistical analysis, with supervisory input from KA-R. RZ, EB, and PY wrote the manuscript. All authors contributed to the article and approved the submitted version.

## Funding

This work was supported by the Biotechnology and Biological Sciences Research Council (BBSRC) and the Diet and Health Research Industry Club (DRINC) (Research Grant BB/N021185/1).

## Conflict of Interest

The authors declare that the research was conducted in the absence of any commercial or financial relationships that could be construed as a potential conflict of interest.

## Publisher's Note

All claims expressed in this article are solely those of the authors and do not necessarily represent those of their affiliated organizations, or those of the publisher, the editors and the reviewers. Any product that may be evaluated in this article, or claim that may be made by its manufacturer, is not guaranteed or endorsed by the publisher.

## Supplementary Material

Supplementary Figure 1 Flow cytometer analysis of main sample and control tubes. In this particular sample, **(B)** the final total number of PS+EVs were obtained by Annexin V+EV numbers displayed in main sample **(A)** subtracting particle numbers displayed in Annexin V negative control; **(C)** A cut-off as CD41+ particles to 1% was set on PE vs. SSC plot for CD41 isotype control, and **(D)** then Annexin V+EVs coexpressing CD41+ displayed in the main sample tube were identified as Annexin V+/CD41+EVs (PDEVs); **(E)** A cut-off as CD105+ particles to 1% was set on APC vs. PB quadrant plot for CD105 isotype control, and **(F)** then Annexin V+EVs coexpressing CD105+ displayed in the main sample tube were identified as Annexin V+/CD105+EVs (EDEVs). APC, Allophycocyanin; EDEVs, endothelial-derived extracellular vesicles; PB, Pacific Blue; PDEVs, platelet-derived extracellular vesicles; PE, phycoerythrin; PS+EVs, phosphatidylserine extracellular vesicles.

Supplementary Figure 2 Tissue factor-induced clot formation and tPA-induced fibrinolysis in PFP. **(A)** Representative images of coagulation process and fibrinolysis; **(B)** Plot of fibrin clot growth vs. time was constructed to calculate coagulation-related parameters such as lag time as the first time for the detection of the significant levels of fibrin clot, the rate of clot growth as the propagation stage of clotting on the interval 15–25 min after the beginning of clot growth, the clot size at the 30th min and clot density as amount of light scattering from a fibrin clot; **(C)** Plot of fibrin clot intensity vs. time was constructed to calculate fibrinolysis-related parameters, such as lysis onset time as the time, when the light scattering intensity (green line) in the clot reach to 30% reduction from the beginning and the lysis progression as the linear rate of the light scattering intensity decrease as the percentage of the initial value in the following 5 min. PFP, platelet-free plasma.
