# Supplementary material for: Circulating Extracellular Vesicles Are Strongly Associated With Cardiovascular Risk Markers
Source: Front Cardiovasc Med. 2022 May 26;9:907457. doi: 10.3389/fcvm.2022.907457 (PMC9178174; doi:10.3389/fcvm.2022.907457)
Supplement: Supplementary Figure 1 — Flow cytometer analysis of main sample and control tubes. In this particular sample, (B) the final total number of PS+EVs were obtained by Annexin V+EV numbers displayed in main sample (A) subtracting particle numbers displayed in Annexin V negative control; (C) A cut-off as CD41+ particles to 1% was set on PE vs. SSC plot for CD41 isotype control, and (D) then Annexin V+EVs coexpressing CD41+ displayed in the main sample tube were identified as Annexin V+/CD41+EVs (PDEVs); (E) A cut-off as CD105+ particles to 1% was set on APC vs. PB quadrant plot for CD105 isotype control, and (F) then Annexin V+EVs coexpressing CD105+ displayed in the main sample tube were identified as Annexin V+/CD105+EVs (EDEVs). APC, Allophycocyanin; EDEVs, endothelial-derived extracellular vesicles; PB, Pacific Blue; PDEVs, platelet-derived extracellular vesicles; PE, phycoerythrin; PS+EVs, phosphatidylserine extracellular vesicles. [file Data_Sheet_1.pdf]

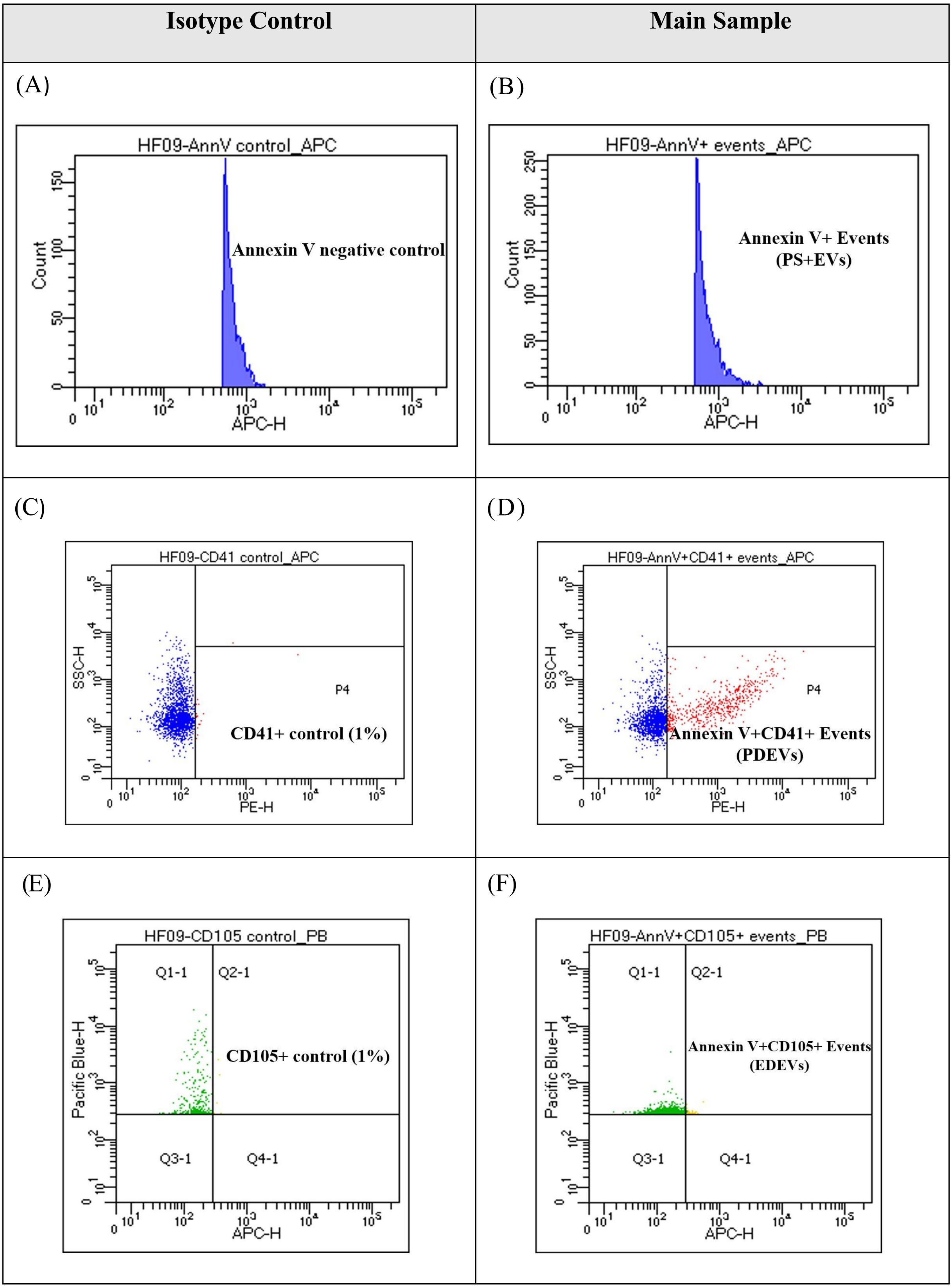

Supplementary _Figure 1. Flow cytometer analysis of main sample and control tubes. In this particular sample, the final total number of PS+EVs were obtained by Annexin V+EV numbers displayed in main sample (**B**) subtracting particle numbers displayed in Annexin V negative control (**A**); A cut-off as CD41+ particles to 1% was set on PE vs SSC plot for CD41 isotype control (**C**), and then Annexin V+EVs coexpressing CD41+ displayed in the main sample tube were identified as Annexin V+/CD41+EVs (PDEVs) (**D**); A cut-off as CD105+ particles to 1% was set on APC vs PB quadrant plot for CD105 isotype control (**E**), and then Annexin V+EVs coexpressing CD105+ displayed in the main sample tube were identified as Annexin V+/CD105+EVs (EDEVs) (**F**). *APC, Allophycocyanin; EDEVs, endothelial-derived extracellular vesicles; PB, Pacific Blue; PDEVs, platelet-derived extracellular vesicles; PE, phycoerythrin; PS+EVs, phosphatidylserine extracellular vesicles.*

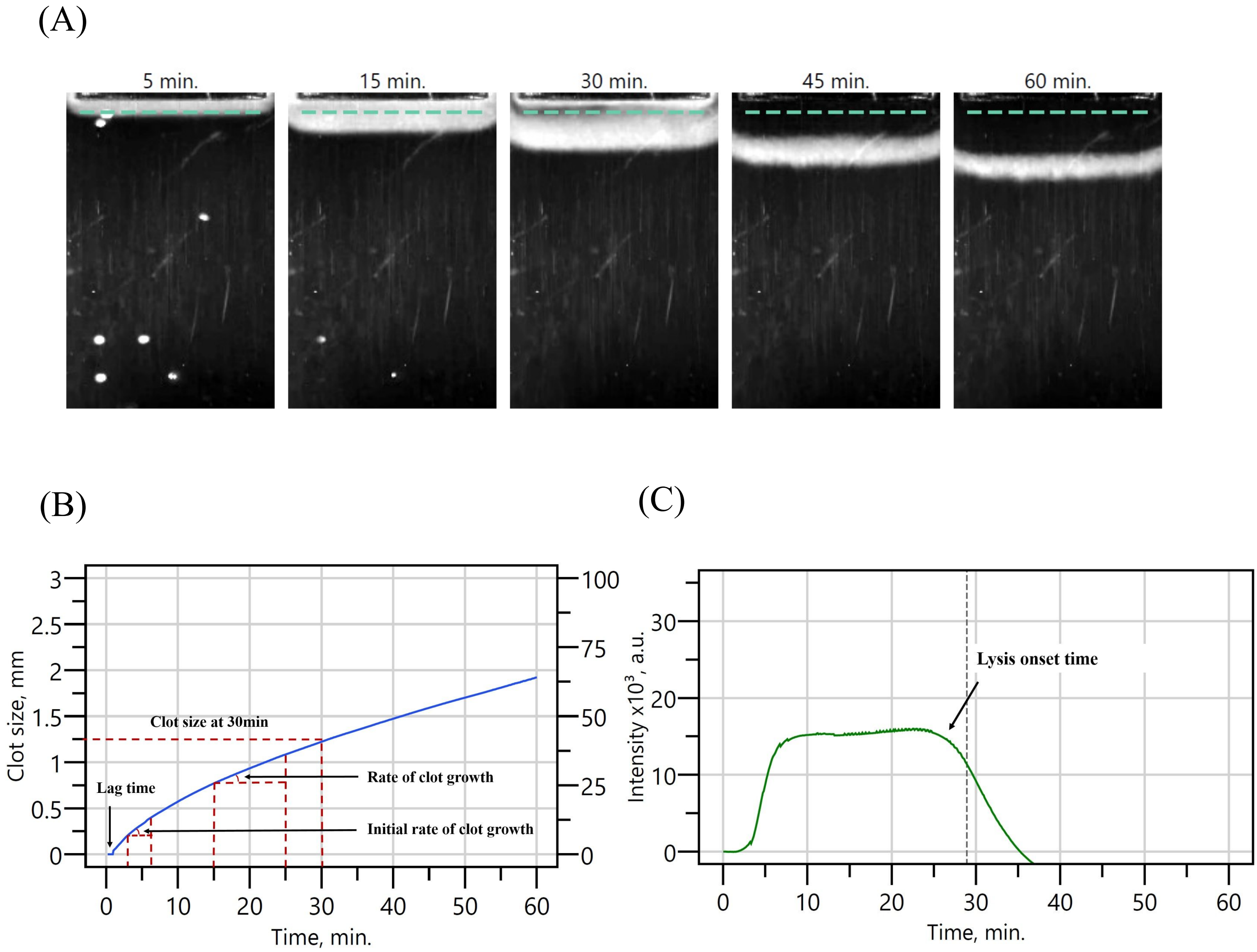

Supplementary_Figure 2. Tissue factor-induced clot formation and tPA-induced fibrinolysis in PFP. (**A**) Representative images of coagulation process and fibrinolysis; (**B**) Plot of fibrin clot growth versus time was constructed to calculate coagulation-related parameters such as lag time as the first time for the detection of the significant levels of fibrin clot, the rate of clot growth as the propagation stage of clotting on the interval 15-25 minutes after the beginning of clot growth, the clot size at the 30th minute and clot density as amount of light scattering from a fibrin clot; (**C**) Plot of fibrin clot intensity versus time was constructed to calculate fibrinolysis-related parameters, such as lysis onset time as the time, when the light scattering intensity (green line) in the clot reach to 30% reduction from the beginning and the lysis progression as the linear rate of the light scattering intensity decrease as the percentage of the initial value in the following 5 minutes. *PFP, platelet-free plasma.*
