# Supplementary material for: Circulating Extracellular Vesicles Are Strongly Associated With Cardiovascular Risk Markers
Source: Front Cardiovasc Med. 2022 May 26;9:907457. doi: 10.3389/fcvm.2022.907457 (PMC9178174; doi:10.3389/fcvm.2022.907457)
Supplement: Supplementary Figure 2 — Tissue factor-induced clot formation and tPA-induced fibrinolysis in PFP. (A) Representative images of coagulation process and fibrinolysis; (B) Plot of fibrin clot growth vs. time was constructed to calculate coagulation-related parameters such as lag time as the first time for the detection of the significant levels of fibrin clot, the rate of clot growth as the propagation stage of clotting on the interval 15–25 min after the beginning of clot growth, the clot size at the 30th min and clot density as amount of light scattering from a fibrin clot; (C) Plot of fibrin clot intensity vs. time was constructed to calculate fibrinolysis-related parameters, such as lysis onset time as the time, when the light scattering intensity (green line) in the clot reach to 30% reduction from the beginning and the lysis progression as the linear rate of the light scattering intensity decrease as the percentage of the initial value in the following 5 min. PFP, platelet-free plasma. [file Data_Sheet_2.pdf]

Supplementary_Table 1. Subjects EVs-related characteristics

EVs Characteristics	All Subjects (n=40)
**EV numbers**	
TEVs(per ml blood) (NTA)	3.6×10^{10} (3.3×10^{10})
EVs Mean Size (nm)	98.0±1.8
EVs Mode Size (nm)	73.7±1.3
PS+EVs (per ml blood) (FCM)	$3.5\times10^{7}\pm2.3\times10^{6}$
PDEVs (per ml blood) (FCM)	$2.2\times10^{7}\pm1.2\times10^{6}$
EDEVs (per ml blood) (FCM)	1.1×10^{6} (1.1×10^{6})
**EVs-induced thrombin generation**	
Lag time for thrombin generation (min)	20.9±0.7
Peak thrombin concentration (nM)	73.6±6.2
Time to peak thrombin concentration(min)	36.6±1.3
Velocity index	5.8±0.7
ETP (nM thrombin × min)	1581.5±140.0

Normally distributed data are shown as mean ± SEM and non-normally distributed data as median (inter-quartile range). *EDEVs, endothelial-derived extracellular vesicles; ETP, endogenous thrombin potential; EVs, extracellular vesicles; FCM, flow cytometry; HDL-C, high-density lipoprotein cholesterol; NTA, nanoparticle tracking analysis; PDEVs, platelet-derived extracellular vesicles; PS+EVs, phosphatidylserine positive extracellular vesicles; TEVs, total extracellular vesicles.*

Supplementary_Table 2. Associations between numbers/size of EVs and conventional cardiovascular risk markers

	Correlations											
			**Age**	**BMI (kg/m^2)**	**SBP (mmHg)**	**DBP (mmHg)**	**TC (mmol/L)**	**TAG (mmol/L)**	**HDL-C (mmol/L)**	**TC/HDL-C ratio**	**Glucose (mmol/L)**	**Risk (%)**
**NTA**	**TEVs/ml blood**	*r*	-.136	**.601****	**.358***	**.550****	-.037	**.655****	-.267	.186	.219	**.559****
		p	.404	**<.001**	**.023**	**<.001**	.822	**<.001**	.096	.252	.174	**<.001**
**FCM**	**PS+EVs/ml blood**	*r*	-.052	.257	.115	.208	.258	.227	-.018	.206	.256	.189
		p	.750	.109	.481	.198	.108	.160	.910	.203	.111	.243
	**PDEVs/ml blood**	*r*	-.014	-.144	-.042	.054	**.330***	.037	.031	.245	.162	.068
		p	.932	.376	.795	.740	**.038**	.821	.849	.128	.319	.675
	**EDEVs/ml blood**	*r*	.018	-.132	.216	.230	-.126	.029	.024	-.098	.124	.254
		p	.914	.418	.181	.153	.440	.859	.885	.548	.444	.114
**NTA**	**Mean size (nm)**	*r*	.168	**-.316***	-.162	-.302	.105	**-.395***	.181	-.097	-.247	.163
		p	.301	**.047**	.319	.058	.519	**.012**	.264	.553	.125	.314
	**Mode size (nm)**	*r*	.110	-.220	-.226	-.239	.135	-.121	.158	-.052	-.146	.285
		p	.498	.173	.162	.138	.407	.457	.331	.751	.367	.074

Correlation significant at the *0.05 or **0.01 level (2-tailed Pearsons correlation). *BMI, body mass index; DBP, diastolic blood pressure; EDEV, endothelial-derived extracellular vesicles; FCM, flow cytometry; HDL-C, high-density lipoprotein cholesterol; NTA, nanoparticle tracking analysis; PDEVs, platelet-derived extracellular vesicles; PS+EVs, phosphatidylserine positive extracellular vesicles; SBP, systolic blood pressure; TAG, triacylglycerol; TC, total cholesterol; TEVs, total extracellular vesicles.*

Supplementary_Table 3. Associations between numbers/size of EVs and thrombogenic markers

			Correlations									
			Thrombin generation					Clot formation				
			Lag time for thrombin generation (min)	Peak thrombin concentration (nM)	Time to peak thrombin concentration (min)	Velocity index (nM thrombin/ min)	ETP (nM thrombin × min)	Rate of clot growth (µm /min)	Lag time (min)	Initial rate of clot growth (µm/min)	Clot size at 30 min (µm)	Clot density
NTA	TEVs /ml blood	*r*	**-.382***	**.588****	**-.453***	**.578****	**.563***	**.569****	.024	.249	**.481****	-.001
		p	**.015**	**<.001**	**.003**	**<.001**	**<.001**	**<.001**	.884	.121	**.002**	.997
FCM	PS+EVs /ml blood	*r*	-.051	.166	-.037	.138	.133	.088	.147	-.065	.082	.168
		p	.752	.305	.821	.396	.414	.589	.366	.689	.614	.299
	PDEVs /ml blood	*r*	.150	-.091	.080	-.059	-.113	.083	-.005	-.074	.069	.025
		p	.356	.577	.624	.716	.488	.610	.974	.652	.671	.877
	EDEVs /ml blood	*r*	-.211	.113	-.210	.139	.104	.292	.081	.123	.282	-.020
		p	.192	.488	.194	.392	.521	.068	.619	.450	.078	.904
NTA	Mean size (nm)	*r*	.040	-.011	.110	-.074	.013	-.175	-.046	**-.330***	-.235	.116
		p	.806	.947	.498	.652	.935	.281	.780	**.038**	.144	.477
	Mode size (nm)	*r*	-.161	.238	-.079	.152	.248	.159	-.018	-.105	.019	.064
		p	.320	.139	.630	.350	.123	.326	.912	.519	.906	.697

Supplementary_Table 3. Associations between numbers/size of EVs and thrombogenic markers (continued)

			Correlations						
			Fibrinolysis		Platelet aggregation				
			Lysis onset time	Lysis progression	ADP	CRP-XL	Epinephrine	TRAP-6	U46619
NTA	TEVs /ml blood	*r*	-.046	.105	.152	.060	.093	.183	.093
		p	.776	.519	.349	.712	.566	.259	.568
FCM	PS+EVs /ml blood	*r*	.077	-.071	.026	-.052	.134	.212	.041
		p	.635	.661	.872	.752	.410	.190	.803
	PDEVs /ml blood	*r*	.008	.011	-.099	-.065	.249	-.026	-.065
		p	.962	.945	.543	.691	.121	.876	.692
	EDEVs /ml blood	*r*	**-.420****	**.469****	.049	-.069	.130	.206	.033
		p	**.007**	**.002**	.764	.671	.423	.202	.838
NTA	Mean size (nm)	*r*	-.158	-.030	-.029	.290	**.314***	-.005	**-.338***
		p	.330	.854	.861	.069	**.048**	.977	**.033**
	Mode size (nm)	*r*	-.170	.092	-.076	.277	.214	-.002	**-.382***
		p	.295	.572	.640	.084	.184	.991	**.015**

Correlation significant at the *0.05 or **0.01 level (2-tailed Pearsons correlation). *ADP, adenosine diphosphate; CRP-XL, cross-linked collagen-related peptide; EDEV, endothelial-derived extracellular vesicles; ETP, endogenous thrombin potential; FCM, flow cytometry; NTA, nanoparticle tracking analysis; PDEVs, platelet-derived extracellular vesicles; PS+EVs, phosphatidylserine positive extracellular vesicles; TEVs, total extracellular vesicles; TRAP-6, thrombin receptor-activated peptide 6.*

Supplementary_Table 4. Association between TEV numbers and 10-year CVD risk score predicted by QRISK2

TEVs	Q1 (10.00-10.36) n=10	Q2 (10.37-10.56) n=13	Q3 (10.57-10.75) n=10	Q4 (10.76-11.61) n=7	*p*-value
**Unadjusted**	11.3% (9.5-13.1)	11.5% (9.9-13.1)	14.2% (12.4-16.1)	16.1% (14.0-18.3)	***p*=0.002**
**Adjusted for age**	10.9% (9.3-12.5)	11.6% (10.2-13.0)	14.7% (13.1-16.3)	15.9% (14.0-17.8)	***p*<0.001**
**Adjusted for age and TAG**	11.2% (9.3-13.0)	11.7% (10.2-13.1)	14.7% (13.0-16.3)	15.4% (12.9-17.9)	***p*<0.001**
**Adjusted for age, TAG and HDL-C**	11.3% (9.5-13.2)	11.7% (10.3-13.1)	14.6% (12.9-16.2)	15.2% (12.7-17.7)	***p*<0.001**

10-year CVD risk (QRISK2 score) against quartiles of TEV numbers. Means with 95% confidence interval, unadjusted or adjusted for variables, as shown. TEV numbers and HDL-C concentration were log-transformed to achieve normal distribution before analysis. *CVD, Cardiovascular diseases; HDL-C, high-density lipoprotein cholesterol; TAG, triacylglycerol; TEVs, total extracellular vesicles.*
